# Association of an *NFKB1* intron SNP (rs4648068) with gastric cancer patients in the Han Chinese population

**DOI:** 10.1186/1471-230X-12-87

**Published:** 2012-07-10

**Authors:** Renquan Lu, Xiang Gao, Yin Chen, Jian Ni, Yongfu Yu, Sheng Li, Lin Guo

**Affiliations:** 1 Department of Clinical Laboratory, Shanghai Cancer Center, Fudan University, Shanghai, 200032, China; 2 Department of Oncology, Shanghai Medical College, Fudan University, Shanghai, 200032, China; 3 Institute of Micro-Nano Science and Technology, Shanghai Jiao Tong University, Shanghai, 200240, China; 4 Department of Biostatistics, School of Public Health, Fudan University, Shanghai, 200032, China; 5 Department of Biochemistry, Dalian Medical University, Shandong, 116011, China; 6 Yulong Biomedical Group, Shanghai, 200233, China

**Keywords:** NFKB1, Gastric cancer, Single nucleotide polymorphism (SNP), Susceptibility

## Abstract

**Background:**

Hyperactivation of nuclear factor-κB (NF-κB) is associated with various types of tumors. This study investigated the susceptibility of the rs4648068 A/G genotype in the intron region of *NFKB1* to gastric cancer and the association of this polymorphism with clinicopathologic variables in gastric cancer patients.

**Methods:**

A hospital-based case–control study of 248 gastric cancer patients and 192 control individuals was conducted in Fudan University Shanghai Cancer Center (Shanghai, China). Single nucleotide polymorphism (SNP) rs4648068 genotype in *NFKB1* from blood samples of a total of 440 people was analyzed by polymerase chain reaction-based genotyping.

**Results:**

The frequencies of the AA, AG, and GG genotypes of the rs4648068 polymorphism were 31.5%, 47.2%, and 21.3% in the gastric cancer patients and 29.7%, 59.9%, and 10.4% in the control individuals, respectively. We found that the GG genotype was associated with a significantly increased risk of gastric cancer (*P* = 0.042). Furthermore, among the gastric cancer cases, the rs4648068 GG genotype was associated with high clinical stage (AOR = 2.27, 95% CI: 1.11- 4.66), lymph node involvement (AOR = 2.90, 95% CI = 1.40- 6.03) and serosa invasion (AOR = 2.78, 95% CI = 1.34- 5.75). However, rs4648068 genotypes were not associated with tumor differentiation in gastric cancer patients.

**Conclusions:**

Homozygous rs4648068 GG was associated with an increased risk of gastric cancer, especially for the lymph node status and serosa invasion in Han Chinese population.

## Background

Gastric cancer (GC) is the second leading cause of cancer-related deaths and the fourth most frequent cancer in the world [1]. Generally, GC mortality is ranked the highest in Eastern Asia (China, Japan), Eastern Europe, and South America compared with other cancerous tumors, whereas GC mortality is ranked the lowest in North America and most parts of Africa. The etiology of this cancer is multifactorial, involving both genetic and environmental risk factors. To clarify the genetic background of the gastric cancer, it is necessary to specifically identify the genetic factors such as single nucleotide polymorphisms (SNPs).

Nuclear factor kappa B (NF-κB) is a family of structurally related eukaryotic transcription factors that are persistently active in the pathogenesis of numerous malignancies, including prostate [2], breast [3,4], colorectal [5], pancreatic [6], oral [7], and gastric carcinoma cancer [8]. The NF-κB family contains five members in mammals: *NFKB1* encoding p50, *NFKB2* encoding p52, *RELA* encoding p65, *REL* encoding c-Rel, and *RELB* encoding Rel-B. The most common dimer is the p65/p50 heterodimer. The human *NFKB1* gene is located at chromosome 4q24. Several groups have shown that there is a relationship between gastric cancer and the polymorphisms of *NFKB1* promoter regions [9,10], and local tumor growth and lymph node spread in gastric cancer have also been proven to be associated with N-myc downstream-regulated genes in previous reports [11-13]. This study investigated the possible association between the SNP polymorphisms of *NFKB1* and susceptibility to gastric cancer and its tumor behavior.

We first screened SNPs throughout the entire *NFKB1* gene locus and selected 4 SNP sites from 99 SNP sites that were in linkage disequilibrium (LD) in the Hapmap CHB (Han Chinese in Beijing, China) data: rs12509517 and rs4648068, which are in LD with the tagging SNP rs230539 (r^2^ > 0.8), and rs4648065 and rs4648037 (r^2^ < 0.8). The genetic associations of the SNPs were examined by comparing the genotypes of 30 unrelated gastric cancer patients with those of 30 healthy controls in a small-scale genotyping study. Then, we conducted a large-scale case–control study on the Han Chinese population to investigate the association of the rs4648068 polymorphism with gastric cancer and clinicopathologic variables of gastric cancer patients using quantitative real-time PCR (qRT-PCR).

## Methods

### Patients and tissue samples

A total of 440 unrelated Chinese participants including 248 patients with gastric cancer and 192 unaffected control participants were recruited with written informed consent, and all human samples were collected with the approval of the Ethics Committee at the Fudan University Cancer Hospital. All subjects were part of the Han population in China. Age, gender, and other clinicopathologic characteristics were evaluated by reviewing medical charts and pathologic records. There were no differences in age and gender between gastric cancer patients and the control participants (*P* > 0.05).

### Sample preparation and *NFKB1* genotyping

DNA samples were prepared and genotyped as described previously [14,15]. Briefly, genomic DNA was extracted from peripheral blood samples of each study subject using the TIANamp Blood DNA Kit (Tiangen Biotech, China). Four *NFKB1* SNPs (rs12509517, rs4648068, rs4648065, rs4648037) were genotyped by SYBR allelic discrimination, and their corresponding qRT-PCR specific primers are listed in Table 1. PCRs were run in a 20 μL reaction solution containing 30 ng of template DNA and 10 μL SYBR Premix Ex Taq (Takara, Japan) according to the manufacturer’s instructions. PCR was performed at 95°C for 10 min and 40 cycles at 95°C for 30 s and 58°C for 30 s. The samples were amplified, read, and analyzed in Opticon® Fluorescence Temperature Cycler (MJ Research, Canada).

**Table 1:** Primers used for quantitative real-time PCR assays

**SNP**	**Primer**	**Primer sequence**
rs12509517	Upstream fwd	5’ ACG TAC TAG GAA GTC CTA C 3’
	Upstream fmut	5’ ACG TAC TAG GAA GTC CTA G 3’
	Downstream rev	5’ GGT TAT GCA GGA TGT TAC CAT TGG 3’
rs4648065	Upstream fwd	5’ TAA CGT ATG CA A CAG GAA C 3’
	Upstream fmut	5’ TAA CGT ATG CA A CAG GAA T3’
	Downstream rev	5’ GAT ATC TTT CTG CAC CTA GGA CTG3’
rs4648068	Upstream fwd	5”TAA TTG TTA GAG ATT CCA A 3’
	Upstream fmut	5’ TAA TTG TTA GAG ATT CCA G3’
	Downstream rev	5’ ACA ATG TTA GAT TTT ACC ATG ATT3’
rs4648037	Upstream fwd	5’ TAA CAC CTT AAA AGG GTG C3’
	Upstream fmut	5’ TAA CAC CTT AAA AGG GTG T3’
	Downstream rev	5’ ATC ATT TAA TCA GTT GCC ATT GGG3’

### Statistical analysis

The Haploview 4.2 software (http://www.broad.mit.edu/mpg) was used to draw a LD map for 99 SNPs (with minor allele frequency ≥ 0.05) located in the *NFKB1* region according to the genotype data for Han Chinese populations of the International HapMap Project (HapMap Data Rel 27 Phase II + III, Feb09, on NCBI B36 assembly, dbSNP b126) and to measure the pairwise LD values between SNPs. The SNPs that were tagged by these 4 SNPs with r^2^ > 0.6 can be illustrated with the LD map [16].

Adjusted odds ratio (AOR) and associated 95% confidence interval (CI) were calculated using multivariate logistic regression analysis. The comparison of age and gender were performed by student *t-* test and Pearson chi-square test, respectively, where *P* < 0.05 indicated a significant difference. All of the association calculations were conducted with SPSS (version 13.0; SPSS Inc., Chicago).

## Results

### Gastric cancer susceptibility association of 4 *NFKB1* SNPs

To identify the genetic variations in *NFKB1* that are associated with a susceptibility to gastric cancer, we examined the *NFKB1* region and selected 4 SNP sites from 99 SNP sites in LD of *NFKB1*: rs12509517 and rs4648068, which are in LD with the tagging SNP rs230539 (r^2^ > 0.8), and rs4648065 and rs4648037 (r^2^ < 0.8). According to the genotype data for the CHB by the International HapMap Project, the values of LD between these 4 SNPs and other ungenotyped polymorphisms in the HapMap database are shown in Additional file: 1 Figure S1, in which rs4648037, rs4648065, rs4648068, and rs12509517 are located at sites 142, 168, 169, and 188, respectively. As illustrated in this Additional file: 1 Figure S1, all of the ungenotyped polymorphisms were tagged by these 4 tag SNPs with r^2^ > 0.6.

A small-scale qRT-PCR genotyping study was conducted to examine the genetic association of these SNPs with gastric cancer. Because polymorphisms in intron or exon regions are all reported to regulate transcription and translocation [17-20], we selected 2 intron polymorphisms (rs12509517, rs4648068) and 2 exon SNPs (rs4648065, rs4648037) out of the SNP pool for our analysis.

Comparison of 30 gastric cancer patients and 30 healthy control individuals showed that only SNP rs4648068 had a significant association with susceptibility to gastric cancer (*P* = 0.029), whereas the other 3 SNPs showed no association with susceptibility to gastric cancer (Table 2). Of the individuals with gastric cancer, 33.3% had the GG genotype in rs4648068, compared with 10% with the GG genotype in the healthy control individuals. In contrast, the probability of AA alleles in gastric cancer patients is 13.3% less than that in healthy controls. Surprisingly, this SNP, rs4648068, is in the intron region of the *NFKB1* gene. Because there are many previous reports on intronic SNPs that were associated with the activity of protein expression [21], diseases [22], or disorders [23] through gene regulation and transcript processing [24], we conducted a large-scale study of rs4648068.

**Table 2:** Genotype distribution of 4 SNPs in gastric cancer patients and control subjects

	**Cases (*n*** **= 30)**		**Health Controls (*n*** **= 30)**		**AOR (95%CI)**	***P*Value**
	**n**	**%**	**n**	**%**		
rs12509517						
GG	14	46.7	9	30.0	1.08(0.53-2.21)	0.601
CG	12	40.0	18	60.0	0.76(0.31-1.63)	0.420
CC	4	13.3	3	10.0	1.00	
rs4648065						
TT	6	20.0	3	10.0	1.73(0.83-4.12)	0.313
CT	8	26.7	3	10.0	1.62(0.99-2.69)	0.065
CC	16	53.3	24	80.0	1.00	
rs4648068						
GG	10	33.3	3	10.0	2.90(1.01-6.93)	0.029*
AG	16	53.4	17	56.7	1.68(0.62-4.01)	0.293
AA	4	13.3	10	33.3	1.00	
rs4648037						
TT	15	50.0	22	73.3	0.63(0.28-1.39)	0.193
CT	8	26.7	5	16.7	0.67(0.40-1.83)	0.390
CC	7	23.3	3	10.0	1.00	

AOR, adjusted odds ratio, which was calculated using multivariate logistic regression adjusted by age and gender; CI, confidence interval.

### An *NFKB1* polymorphism is associated with an increased risk of gastric cancer

After the preliminary small-scale genotyping, we conducted a large-scale genotyping study of rs4648068. Analysis of the rs4648068 genotype polymorphism showed a significant difference between gastric cancer patients and control individuals (Table 3). The rs4648068 GG genotype was significantly more common in the gastric cancer patients than in the control individuals (*P* = 0.042), which further implied that homozygous GG was a genetic risk factor for gastric cancer and agreed well with the results of our preliminary small-scale genotyping study described above. Results of stratified analyses by age, clinical stage, tumor differentiation, lymph node status, and serosa invasion with the *NFKB1* rs4648068 A/G polymorphism variant genotypes and alleles are presented in Table 4.

**Table 3:** **Genotype distribution and allelic frequencies of*NFKB1*rs4648068 polymorphism in gastric cancer patients and control subjects**

	**Gastric Cancer**	**Controls**	**AOR (95%CI)**^**Δ**^	***P*value**^**Δ**^
Cases (*n*)	248	192	/	/
Age	57 ± 11*	53 ± 15	/	/
Gender (M/F)	160/88**	119/73	/	/
Genotype				
GG(%)	53(21.3)^#^	20(10.4)	1.92(1.02-3.60)	**0.042**
AG(%)	117(47.2)	115(59.9)	0.75(0.48-1.16)	0.195
AA (%)	78(31.5)	57(29.7)	1.00	
Allelic frequencies				
G(%)	223(45.0)	155(40.4)	1.13(0.99-1.36)	0.198
A(%)	273(55.0)	229(59.6)	1.00	

* and **, *P* > 0.05 and minor allele frequency (MAF) = 0.346; M, male; F, female; ^#^, *P* = 0.042 (GG is risk factor); ^**Δ**^**,** multivariate logistic regression adjusted by age and gender.

**Table 4:** **Association between the genotype of the*NFKB1*rs4648068 polymorphism and patient characteristics**

	**N**	**GG**	**AOR (95%CI)**^**Δ**^	***P*value**^**Δ**^	**GA**	**AOR (95%CI)**^**Δ**^	***P*value**^**Δ**^	**AA**	**AOR (95%CI)**^**Δ**^***P*value**^**Δ**^	**G**	**A**	AOR (95%CI)*	*P*value*
Clinical Stage													
III + IV	128	33(25.8)	2.27(1.11 ~ 4.66)	0.025	62(48.4)	1.55(0.87 ~ 2.78)	0.139	33(25.8)	-	128(50.0)	128(50.0)	1.86(1.22 ~ 2.82)	0.004
I + II	120 20(16.7)	-			55(45.8)	-		45(37.5)	-	95(39.6)	145(60.4)	-	
Tumor differentiation													
Poor	118	29(24.6)	1.34(0.66-2.70)	0.418	52(44.1)	0.89(0.50 ~ 1.58)	0.680	37(31.4)	-	110(46.6)	126(53.4)	1.08(0.71 ~ 1.63)	0.729
Well & moderate	130	24(18.5)	-		65(50.0)	-		41(31.5)	-	113(43.5)	147(56.5)	-	
Lymph node status													
Positive	111	29(26.1)	2.90 (1.40 ~ 6.03)	0.004	59(53.2)	2.44(1.33-4.50)	0.004	23(20.7)	-	117(52.7)	105(47.3)	2.67(1.72 ~ 4.13)	0.001
Negative	137	24(17.5)	-		58(42.3)	-		55(40.1)		106(38.7)	168(61.3)	-	
Serosa invasion													
Positive	126	34(27.0)	2.78(1.34 ~ 5.75)	0.006	62(49.2)	1.74(0.97-3.13)	0.064	30(23.8)	-	130(51.6)	122 (48.4)	2.17(1.42 ~ 3.31)	0.001
Negative	122	19(15.6)	-		55(45.1)			48(39.3)	-	93(38.1)	151 (61.9)	-	

^Δ^, Multivariate logistic regression adjusted by age and gender (genotype AA, AOR = 1.00); *, multivariate logistic regression adjusted by age and gender (A carrier, AOR = 1.00).

### Association between *NFKB1* rs4648068 polymorphism and age/gender of gastric cancer patients

The role of the rs4648068 polymorphism in different age groups and gender groups of gastric cancer patients was evaluated. Because age and gender are reported to be the 2 major risk factors of gastric cancer, and this disease is reported to be more common in men over the age of 55 than in other groups [25-28], we divided the gastric cancer patients into 2 age groups (age ≤ 55 and age > 55), or groups that were divided by gender. However, the associations of this polymorphism with age and gender were not statistically significant.

### Association of *NFKB1* rs4648068 polymorphism and clinical stage of gastric cancer

We evaluated whether the association between the rs4648068 polymorphism and gastric cancer was modified by the clinical stages of the cancer by dividing the gastric cancer patients into 2 groups: clinical stages I + II and clinical stages III + IV. The frequency of the GG genotype in clinical stages III + IV was significantly higher than that in clinical stages I + II (*P* = 0.025, AOR = 2.27, 95% CI: 1.11- 4.66), indicating an association between high clinical stage and the G allele (Table 4).

### *NFKB1* rs4648068 is associated with the survival of gastric cancer patients

As indicated in Table 4, lymph node status and serosa invasion were significantly associated with the survival of gastric cancer patients. Stratified analyses by lymph node status showed that patients carrying the G allele were more likely than those carrying the A allele to have a positive lymph node status (52.7% *versus* 38.7%, *P* = 0.001). Furthermore, there was a significant correlation between the genotype and serosa invasion. The frequency of the GG genotype in serosa invasion-positive patients was significantly higher than that in serosa invasion-negative patients (27.0% *versus* 15.6%, *P* = 0.006). The frequency of G carriers in serosa invasion-positive patients was also significantly higher than that in serosa invasion-negative patients (51.6% *versus* 38.1%, *P* = 0.001, AOR = 2.17, 95% CI: 1.42- 3.31). However, rs4648068 genotypes were not associated with the tumor differentiation in gastric cancer patients (Table 4).

## Discussion

NF-κB was reported to be a proinflammatory transcription factor, which could bind the enhancer of the kappa light chain of immunoglobulin and promote tumorigenesis [29,30]. The rs4648068 (A > G) polymorphism was identified in a study of the analysis of variation in *NFKB1* genes and expression levels of NF-κB regulated molecules, and SNP rs4648068 was subsequently reported to be associated with VCAM1 and LDL phenotypes [31,32].

Considering there is a relationship between gastric cancer and the promoter region of *NFKB1*[9,10] and many reports also showed that the genetic variations in introns might involve alternative gene regulation, transcript processing, or chromosomal rearrangements [16,17,23,24], the association between the rs4648068 polymorphism in *NFKB1* and gastric cancer has been studied. Here, we found that the rs4648068 polymorphism had a significantly different distribution in gastric cancer patients and healthy control individuals in a preliminary small-scale genotyping study (*P* = 0.029). Therefore, we investigated the role of the *NFKB1* rs4648068 polymorphism in gastric cancer susceptibility in the Han Chinese population in a case–control study. The results indicated that people with the homozygous GG alleles in rs4648068 had a 2.65-fold increased risk of gastric cancer compared people carrying other rs4648068 alleles (*P* = 0.042).

Surprisingly, although reports showed that age and gender are the 2 major risk factors of gastric cancer, and gastric cancer is reported to be more common in men over the age of 55 than in other groups of individuals [25-28], our stratified analysis by age and gender did not modify the association between rs4648068 and the risk of gastric cancer. Lewander’s group [33] and Gao’s group [34] have reported that the genetic polymorphisms have the opposite effect in Swedish populations compared with Chinese populations and in Asian populations compared with Caucasian populations. Whether our results are ethnically dependent requires further elucidation. In addition, although the minor allele frequency (MAF) of rs4648068 is high (0.346), this conclusion would be more convincing if it can be replicated in another cohort.

On the basis of our genetic association data in the Han Chinese population, the SNP within an *NFKB1* intron region (rs4648068) showed marginal significance. This suggested that there might be correlation between *NFKB1* genotypes and the gastric cancer clinicopathologic characteristics such as lymph node status and serosa invasion because of the different associations that were observed for the high-risk subsets and the low-risk subsets.

The SNP rs4648068 (A or G) resides in intron 11 of *NFKB1*, occupying position 103,518,305 of chromosome 4. The predominant nucleotide at this position is A for human, chimpanzee, and macaque, but G for orangutan, mouse, and dog. There is no conserved trait at this position in the genome of elephants, opossum, chicken, or zebrafish, although the corresponding region is Nfkb in these organisms. The percentages of the A and G alleles in the Han Chinese ethic group in Beijing are 0.609 and 0.391 (http://www.Hapmap.org), respectively; our study in Shanghai found that the percentages of the A and G alleles are 0.551 and 0.449, respectively. The discrepancy may result from geographic factors because the percentages of A and G alleles in Han Chinese in Denver, Colorado, are also different from the above findings (A: 0.596; G: 0.404, data from http://www.Hapmap.org). To elucidate the potential functional role of SNP rs4648068, we also performed a bioinformatic data mining study using the UCSC genome browser (http://genome.ucsc.edu/cgi-bin/hgTracks) and Encode databases. The region near rs4648068 in intron 11 of *NFKB1* exhibited increased levels of H3K9me1 and H3K4me1 (the highest value of H3K9me1 is 15, while the increase of the H3K4me1 level is not as remarkable as that of H3K9me1). These tracks displayed maps of chromatin state that were generated by the Broad/MGH ENCODE group using chromatin immunoprecipitation coupled with sequencing (ChIP-seq). Chemical modifications (methylation for H3K9me1 and acetylation for H3K4me1) to the histone proteins that are present in chromatin influence gene expression by changing the accessibility of the chromatin to transcription factors. The increased levels of chemo-modification imply that rs4648408 may be located in a regulatory element, such as an enhancer. Hence, the G allele of rs4648068 may alter the power of a potential enhancer, which may have an influence on disease in the host.

In the present study, we observed that the frequency of the G allele in clinical stages III + IV patients was significantly higher than that in clinical stages I + II patients, indicating that this polymorphism is associated with high clinical stage (*P* = 0.025). On the other hand, the stratified analysis by lymph node status showed that patients with the GG genotype and G carriers were more likely than those with the AA genotype and A carriers to have a positive lymph node status (*P* = 0.004 and 0.001, respectively), suggesting that this polymorphism might contribute to the constitutive NF-κB activity in gastric cancer.

Our investigations also demonstrated that the frequency of the GG homozygous_genotype in serosa invasion-positive patients was significantly higher than that in serosa invasion-negative patients (*P* = 0.006). Tumor invasion is regulated by numerous NF-κB target gene products, including MMP-9, TIMP-1/2, PAL 2, CXCR4, interleukin-8 (IL-8), and other chemokines [29,35,36]. NF-κB plays an essential role in the migration and the organ-specific homing of metastatic breast cancer cells [35,37].

### Further study

In our future experiments, we plan to use a dual-luciferase reporter assay in gastric cancer cell lines (MKN28, SNU216, SNU16, SCG7901, and HGC-27) to observe the LPS-induced luciferase expression level, which correlates with the mRNA level, to explore whether the mechanism of this association involves alternative gene regulation and transcript processing.

## Conclusions

In this study, we have shown that people who carry the GG homozygous_genotype of the rs4648068 polymorphism in the *NFKB1* gene appear to be at increased risk for developing gastric cancer. Furthermore, in gastric cancer patients, the G allele is associated with high clinical stage, positive lymph node status, and positive serosa invasion.

## Competing interests

The authors have no competing interests to declare.

## Author contributions

RQL designed the study and analyzed and interpreted the data. XG participated in clinical data and information collection. YC and SHL collected all samples and performed qRT-PCR genotyping experiments. YFY and JN were involved in statistical analysis of the data and drafted and edited the manuscript. LG conceived and supervised the project and reviewed the manuscript. All authors have read and approved the final manuscript.

## Pre-publication history

The pre-publication history for this paper can be accessed here:

http://www.biomedcentral.com/1471-230X/12/87/prepub

## Supplementary Material

Additionaf file 1 **Figure S1**. LD map including 4 SNPs of the *NFKB1* genomic region in the Han Chinese population. The rs4648037, rs4648065, rs4648068, and rs12509517 SNPs are located at sites 142,168,169, and 188, respectively. The values of LD between these 4 SNPs (shown are r^2^ > 0.6) and other ungenotyped polymorphisms are presented. Click here for file
