# Supplementary material for: Association of an NFKB1 intron SNP (rs4648068) with gastric cancer patients in the Han Chinese population
Source: BMC Gastroenterol. 2012 Jul 10;12:87. doi: 10.1186/1471-230X-12-87 (PMC3407756; doi:10.1186/1471-230X-12-87)
Supplement: Additionaf file 1 — Figure S1. LD map including 4 SNPs of the NFKB1 genomic region in the Han Chinese population. The rs4648037, rs4648065, rs4648068, and rs12509517 SNPs are located at sites 142,168,169, and 188, respectively. The values of LD between these 4 SNPs (shown are r2 > 0.6) and other ungenotyped polymorphisms are presented. [file 1471-230X-12-87-S1.doc]

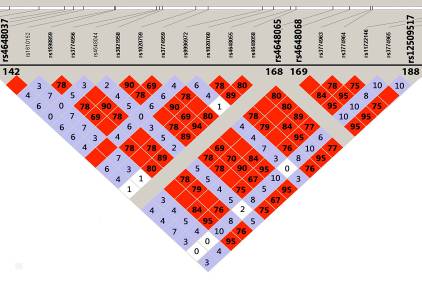


**Supplementary Figure:**

**LD map including 4 SNPs of the *NFKB1* genomic region in the Han Chinese population. The rs4648037, rs4648065, rs4648068, and rs12509517 SNPs are located at sites 142,168,169, and 188, respectively. The values of LD between these 4 SNPs (shown are r^2^ > 0.6) and other ungenotyped polymorphisms are presented.**
